# Traveling Wave Solutions for Epidemic Cholera Model with Disease-Related Death

**DOI:** 10.1155/2014/409730

**Published:** 2014-04-27

**Authors:** Tianran Zhang, Qingming Gou

**Affiliations:** ^1^School of Mathematics and Statistics, Southwest University, Chongqing 400715, China; ^2^College of Mathematics & Computer Science, Yangtze Normal University, Chongqing 408100, China

## Abstract

Based on Codeço's cholera model (2001), an epidemic cholera model that incorporates the pathogen diffusion and disease-related death is proposed. The formula for minimal wave speed *c* ^∗^ is given. To prove the existence of traveling wave solutions, an invariant cone is constructed by upper and lower solutions and Schauder's fixed point theorem is applied. The nonexistence of traveling wave solutions is proved by two-sided Laplace transform. However, to apply two-sided Laplace transform, the prior estimate of exponential decrease of traveling wave solutions is needed. For this aim, a new method is proposed, which can be applied to reaction-diffusion systems consisting of more than three equations.

## 1. Introduction

In the past and at present, cholera has been a serious threat to human health, which is an acute, diarrheal illness caused by infection of the intestine with the bacterium* Vibrio cholera*. An estimated 3–5 million cases and over 100,000 deaths occur each year around the world [1]. The cholera bacterium is usually found in water or food sources that have been contaminated by feces from a person infected with cholera. Cholera is most likely to be found and spread in places with inadequate water treatment, poor sanitation, and inadequate hygiene. Therefore, cholera outbreaks have been occurring in developing countries—for example, Iraq (2007-2008), Guinea Bissau (2008), Zimbabwe (2008-2009), Haiti (2010), Democratic Republic of Congo (2011-2012), and Sierra Leone (2012) [2].

Many mathematical models were proposed to understand the propagation mechanism of cholera, the earlier one of which was established by Capasso and Paveri-Fontana [3] to study the 1973 cholera epidemic in the Mediterranean region as follows:

(1) $$\begin{array}{c} \frac{dI}{dt}=g\left(B\right)-a_{22}I,\\ \frac{dB}{dt}=-a_{11}B+a_{12}I, \end{array}$$

where *B*(*t*) and *I*(*t*) denote the concentrations of the pathogen and the infective populations, respectively. In addition, Codeço [4] investigated the role of the aquatic pathogen in dynamics of cholera through the following susceptible-infective-pathogen mode:

(2) $$\begin{array}{c} \frac{dS}{dt}=n\left(H-S\right)-a\frac{SB}{K+B},\\ \frac{dI}{dt}=a\frac{SB}{K+B}-rI,\\ \frac{dB}{dt}=eI-\left(mb-nb\right)B, \end{array}$$

where *S*(*t*) is the susceptible individuals. In this model, human is divided into two groups: the susceptible and the infective. As pointed out in [4–8], bacterium* Vibrio cholera* can spread by direct human-to-human and indirect environment-to-human modes. To understand the complex dynamics of cholera, model (2) is extended by [6, 9–15], and so forth.

In all previous models, the influences of space distribution of human on the transmission of cholera are omitted. Cholera usually spreads in spacial wave [16]. Cholera bacteria live in rivers and interact with the plankton on the surface of the water [17]. When individuals drink contaminated water and are infected, then they will release cholera bacteria through excretion [18]. Capasso et al. [19–22] developed model (1) by incorporating the bacterium diffusion in a bounded area and studied the existence and stability of solutions. To deeply investigate the interaction of transmission modes and bacterium diffusion, Bertuzzo et al. [23, 24] incorporated patchy structure into model (2) and supposed that the pathogen in water could diffuse among these patches. Furthermore, Mari et al. [25] studied the influence of diffusion of both human and pathogen on cholera dynamics through a patchy model.

Infectious case usually is found firstly at some location and then spreads to other areas. Consequently, the most important question for cholera is as follows: what is the spreading speed of cholera? However, the above spacial models mainly focus on the stability of solutions, not the spreading speed. Traveling wave solution is an important tool used to study the spreading speed of infectious diseases [26–28]. Based on Capasso's model (1), Zhao and Wang [29], Xu and Zhao [30], Jin and Zhao [31], and Hsu and Yang [32] studied the influences of pathogen diffusion on the spread speed of cholera.

In above diffusive cholera models, diffusion of aquatic pathogen is neglected. In this paper, we investigate the effects of the disease-related death and aquatic pathogen in cholera epidemic dynamics by developing model (2). Based on model (2) and ignoring natural birth and death, a general diffusive epidemic cholera model incorporating the disease-related death and aquatic pathogen dynamics can be formulated as the following reaction-diffusion system:

(3) $$\begin{array}{c} \frac{\partial S}{\partial t}=-f\left(B\right)S,\\ \frac{\partial I}{\partial t}=f\left(B\right)S-\delta I,\\ \frac{\partial B}{\partial t}=d\frac{\partial^{2}B}{\partial x^{2}}+\gamma I-mB, \end{array}$$

where *S* = *S*(*x*, *t*) and *I* = *I*(*x*, *t*) denote the concentrations of susceptible and infected individuals, respectively, and *B* = *B*(*x*, *t*) is the concentration of the infectious agents. *δ* is the disease-related death rate, *γ* denotes the contribution of each infected person to the concentration of cholera, and *m* is the net death rate of the vibrio. *f*(*B*) is the environment-to-human transmission incidence. Similar to [15], we assume that *f*(*B*) satisfies

(A1) *f*(0) = 0, lim⁡_*B*→+*∞*_ *f*(*B*) < +*∞*, *f*′(*B*) > 0, −*M*_0_ ≤ *f*′′(*B*) ≤ 0 for *B* ≥ 0.

From hypothesis (A1), we have *f*(*B*) ≤ *f*′(0)*B*.

In this paper, we study the traveling wave solutions of model (3). The formula for minimal wave speed c* is given. To prove the existence of traveling wave solutions for *c* > c*, an invariant cone is constructed and Schauder's fixed point theorem is introduced. Schauder's fixed point theorem is applied widely to prove the existence of traveling wave solutions (e.g., [26, 33, 34]). However, unlike Wang and Wu [34], the cone in our paper is bounded. Motivated by [34–37], we prove the nonexistence of traveling wave solutions for *c* < c* by two-sided Laplace transform, which was firstly introduced to prove the nonexistence of traveling wave solutions by Carr and Chmaj [37] and then was applied by [34–36]. To apply two-sided Laplace transform, the exponential decrease of traveling wave solutions is needed, which is proved in [34] by analysis method. However, it cannot be applied to our model due to the nonlinearity of cholera incidence. Therefore, in this paper, a new method is proposed to get the exponential decrease of traveling wave solutions, which is inspired by the proof of Stable Manifold Theorem in [38]. In addition, our method can be applied to reaction-diffusion systems consisting of more than three equations.

This paper is organized as follows.  Section 2 is focused on the existence of traveling wave solutions. Firstly, the existence of traveling wave solutions for original system is proved to be equivalent to that of a new simple system. Then, two pairs of upper and lower solutions are constructed to get an invariant cone and Schauder's fixed point theorem is applied for new system.  Section 3 is devoted to the nonexistence of traveling wave solutions. For this aim, a new method is proposed to show the exponential decrease of traveling wave solutions and two-sided Laplace transform is used.

## 2. Existence of Traveling Wave Solutions

For convenience in discussing the model, we introduce dimensionless variables and parameters. Setting

(4) $$\begin{array}{c} u_{1}=\frac{m}{\delta }S,\;\;u_{2}=I,\;\;u_{3}=\frac{m}{\gamma }B,\\ \tau =mt,\;\;y=\sqrt{\frac{m}{d}}x, \end{array}$$

we obtain

(5) $$\begin{array}{c} u_{1,\tau }=-g\left(u_{3}\right)u_{1},\\ u_{2,\tau }=\kappa \left[g\left(u_{3}\right)u_{1}-u_{2}\right],\\ u_{3,\tau }=u_{3,yy}+u_{2}-u_{3}, \end{array}$$

where

(6) $$\begin{array}{c} \kappa =\frac{\delta }{m},\;\;g\left(u_{3}\right)=f\frac{\left(\gamma u_{3}/m\right)}{m},\\ u_{i,\tau }=\frac{\partial u_{i}}{\partial \tau },\;\;u_{3,yy}=\frac{\partial^{2}u_{3}}{\partial y^{2}}. \end{array}$$

Obviously, *g*(*u*_3_) also satisfies assumption (A1) with *M*_0_ being replaced by a new constant ${\bar{M}}_{0}$.

A traveling wave solution of system (5) is a nonnegative nontrivial solution of the form

(7) $$\begin{array}{c} u_{1}\left(y,\tau \right)=U\left(\xi \right),\\ u_{2}\left(y,\tau \right)=W\left(\xi \right),\\ u_{3}\left(y,\tau \right)=V\left(\xi \right),\;\;\xi =y+c\tau \end{array}$$

satisfying boundary condition

(8) $$\begin{array}{c} \left(U\left(-\infty \right),W\left(-\infty \right),V\left(-\infty \right)\right)=\left(U^{0},0,0\right),\\ \left(U\left(+\infty \right),W\left(+\infty \right),V\left(+\infty \right)\right)=\left(U^{1},0,0\right), \end{array}$$

where *U* ^0^ > *U* ^1^ ≥ 0.

Before giving the main theorem, we introduce the equation for minimal wave speed:

(9) $$\begin{array}{c} \Delta \left(c\right)=b_{3}c^{6}+b_{2}c^{4}+b_{1}c^{2}+b_{0}=0, \end{array}$$

where

(10) b3=−2κ[2g′(0)U0−1]−1−κ2,b2=−2κ[9g′(0)U0−4]−2κ2−2κ3−6κ2g′(0)U0−4,b1=8κ2−8κ3−κ4−36κ2g′(0)U0 +27κ2(g′(0)U0)2+6κ3g′(0)U0,b0=4κ4[g′(0)U0−1].

The main result of this section is given as follows.

**Theorem 1**

Suppose *U* ^0^ > 1/*g*′(0). Then, there exists a positive constant c* which is the only positive root of (9). For any >c*, system (5) has a traveling wave solution (*U*(*y* + *cτ*), *W*(*y* + *cτ*), *V*(*y* + *cτ*)) satisfying boundary condition (8) such that *U*(*ξ*) is nonincreasing in *R*. Furthermore, one has that

(11) $$\begin{array}{c} \overset{+\infty }{\underset{-\infty }{\int }}W\left(\eta \right)d\eta =\overset{+\infty }{\underset{-\infty }{\int }}V\left(\eta \right)d\eta =c\left(U^{0}-U^{1}\right),\\ 0\leq V\left(\xi \right)\leq U^{0}-U^{1}. \end{array}$$

To study the existence of traveling wave solutions, using constant variation method in the second equation of (5) gives

(12) u2(y,τ)=κ∫−∞τe−κ(τ−ζ)g(u3(y,ζ))u1(y,ζ)dζ=κ∫0+∞e−κηg(u3(y,τ−η))u1(y,τ−η)dη.

Then, system (5) changes into

(13) u1,τ=−g(u3(y,τ))u1(y,τ),u3,τ=u3,yy+κ∫0+∞e−κηg(u3(y,τ−η))u1(y,τ−η)dη −u3(y,τ).

**Lemma 2**

  (*U*(*y* + *cτ*), *V*(*y* + *cτ*)) is a traveling wave solution of system (13) satisfying boundary condition

(14) $$\begin{array}{c} U\left(-\infty \right)=U^{0},\;\;U\left(+\infty \right)=U^{1},\\ V\left(-\infty \right)=V\left(+\infty \right)=0, \end{array}$$

if and only if (*U*(*y* + *cτ*), *W*(*y* + *cτ*), *V*(*y* + *cτ*)) is a traveling wave solution of system (5) satisfying boundary condition (8), where

(15) W(y+cτ) =κ∫0+∞e−κηg(V(y+cτ−cη))U(y+cτ−cη)dη.

**Proof**

Assume (*U*(*y* + *cτ*), *V*(*y* + *cτ*)) is a traveling wave solution of system (13) satisfying boundary condition (14). Obviously, (*U*(*y* + *cτ*), *W*(*y* + *cτ*), *V*(*y* + *cτ*)) is a solution of system (5). To prove the necessity, it is enough to show that *W*(+*∞*) = *W*(−*∞*) = 0. Consider

(16) lim⁡ξ→±∞W(ξ)=lim⁡ξ→±∞κ∫0+∞e−κηg(V(ξ−cη))U(ξ−cη)dη=lim⁡ξ→±∞(κ/c)∫−∞ξeκs/cg(V(s))U(s)dseκξ/c=lim⁡ξ→±∞g(V(ξ))U(ξ)=0,

where the third equality is due to L'Hopital principal. The sufficiency is clear and is omitted. The proof is completed.

From Lemma 2, we only need to study traveling wave solutions of (13) satisfying boundary condition (14). Substituting traveling profile

(17) $$\begin{array}{c} u_{1}\left(y,\tau \right)=U\left(\xi \right),\;\;u_{3}\left(y,\tau \right)=V\left(\xi \right),\;\;\xi =y+c\tau \end{array}$$

into system (13) yields the following equations:

(18) $$\begin{array}{c} {cU}^{\prime }=-g\left(V\left(\xi \right)\right)U\left(\xi \right),\\ {cV}^{\prime }=V^{\prime \prime }+\kappa \overset{+\infty }{\underset{0}{\int }}e^{-\kappa \eta }g\left(V\left(\xi -c\eta \right)\right)U\left(\xi -c\eta \right)d\eta -V\left(\xi \right), \end{array}$$

where ′ denotes the derivative with respect to *ξ*.

In the following, we will use Schauder's fixed point theorem to prove the existence of traveling wave solutions. To achieve this goal, we firstly linearize the second equation of (18) at (*U* ^0^, 0) and obtain

(19) $$\begin{array}{c} c\phi^{\prime }=\phi^{\prime \prime }+\kappa g^{\prime }\left(0\right)U^{0}\overset{+\infty }{\underset{0}{\int }}e^{-\kappa \eta }\phi \left(\xi -c\eta \right)d\eta -\phi \left(\xi \right). \end{array}$$

Substituting *ϕ*(*ξ*) = *e* ^*λξ*^ *v* into (19), we get the characteristic equation

(20) $$\begin{array}{c} \Theta \left(\lambda \right)=\lambda^{2}-c\lambda +\kappa g^{\prime }\left(0\right)U^{0}\overset{+\infty }{\underset{0}{\int }}e^{-(\kappa +c\lambda )\eta }d\eta -1=0; \end{array}$$

that is,

(21) $$\begin{array}{c} H\left(\lambda \right)=\lambda^{3}+a_{2}\lambda^{2}+a_{1}\lambda +a_{0}=0, \end{array}$$

where *a*_0_ = *κ*[*g*′(0)*U* ^0^ − 1]/*c*, *a*_1_ = −(1 + *κ*) < 0, and *a*_2_ = (*κ* − *c* ^2^)/*c*. To investigate distribution of roots of (21), denote

(22) $$\begin{array}{c} p=a_{1}-\frac{a_{2}^{2}}{3},\\ q=\frac{2a_{2}^{3}}{27}-\frac{a_{1}a_{2}}{3}+a_{0},\\ \Delta_{0}=\frac{q^{2}}{4}+\frac{p^{3}}{27}, \end{array}$$

and introduce the following lemma [39].

**Lemma 3**

(a) If Δ_0_ > 0, (21) has one real root and two nonreal complex conjugate roots.

(b) If Δ_0_ = 0, (21) has a multiple root and all its roots are real.

(c) If Δ_0_ < 0, (21) has three distinct real roots.

Then, we get the following lemma about the distribution of eigenvalues.

**Lemma 4**

Assume *U* ^0^ > 1/*g*′(0). Then, there exists a constant c* > 0 which is the only positive root of (9) such that

- if 0 < *c* < c*, (21) has a negative real root and two nonreal complex conjugate roots with positive real parts;
- if *c* = c*, (21) has a negative real root and a positive real multiple root;
- if *c* > c*, (21) has a negative real root and two different positive real roots;
- one can assume *c* > c* and let *λ*_1_ < *λ*_2_ be the two positive roots of (21); then *H*(*λ*_1_ + *ε*) < 0 if 0 < *ε* < *λ*_2_ − *λ*_1_.

**Proof**

Obviously, *b*_3_ < 0, *b*_2_ < 0, and *b*_0_ > 0. By Descartes' rule of signs, Δ(*c*) = 0 has only one positive root c* such that Δ < 0 for *c* > c* and Δ > 0 for 0 < *c* < c*. Direct calculations show that Δ_0_ = Δ/(108*c* ^4^). Since *a*_0_ > 0 and *a*_1_ < 0, Descartes' rule of signs shows that (21) has only one negative real root and Routh-Hurwitz criterion indicates that (21) has roots with positive real parts. Then, the combination of Lemma 3 and above analysis completes the proof of (a)–(c). (d) is obviously true, since *H*(*λ*) is a cubic polynomial.

In this section, we always suppose *U* ^0^ > 1/*g*′(0) and *c* > c* unless other conditions are specified. Denote *λ*_1_ < *λ*_2_ to be the two positive roots of (21) and define

(23) $$\begin{array}{c} \underline{U}\left(\xi \right)=\max\left\{U^{0}-\sigma e^{\alpha \xi },0\right\},\\ \bar{V}\left(\xi \right)=\min\left\{e^{\lambda_{1}\xi },V^{0}\right\},\\ \underline{V}\left(\xi \right)=\max\left\{e^{\lambda_{1}\xi }\left(1-Me^{\varepsilon \xi }\right),0\right\}, \end{array}$$

where *V* ^0^ = *JU* ^0^, *J* = lim⁡_*V*→+*∞*_ *g*(*V*).

**Lemma 5**

The function $\bar{V}(\xi )$ satisfies inequality

(24) $$\begin{array}{c} {c\bar{V}}^{\prime }\geq {\bar{V}}^{\prime \prime }+\kappa U^{0}\overset{+\infty }{\underset{0}{\int }}e^{-\kappa \eta }g\left(\bar{V}\left(\xi -c\eta \right)\right)d\eta -\bar{V}\left(\xi \right) \end{array}$$

for any *ξ* ≠ ln⁡*V* ^0^/*λ*_1_.

**Proof**

Firstly, assume *ξ* < ln⁡*V* ^0^/*λ*_1_ and, therefore, $\bar{V}(\xi )=e^{\lambda_{1}\xi }$. Since $\bar{V}(\xi )$ satisfies (19) and *g*′′(*V*) ≤ 0 for any *V* ≥ 0, we have

(25) cV¯′−V¯′′−κU0∫0+∞e−κηg(V¯(ξ−cη))dη+V¯(ξ)=cV¯′−V¯′′−κg′(0)U0∫0+∞e−κηV¯(ξ−cη)dη+V¯(ξ) −κU02∫0+∞e−κη[g′′(θV¯(ξ−cη))][V¯(ξ−cη)]2dη=−κU02∫0+∞e−κη[g′′(θV¯(ξ−cη))][V¯(ξ−cη)]2dη≥0,

where 0 < *θ* < 1.

Secondly, set *ξ* > ln⁡*V* ^0^/*λ*_1_, which implies $\bar{V}(\xi )=V^{0}$. We have that

(26) cV¯′−V¯′′−κU0∫0+∞e−κηg(V¯(ξ−cη))dη+V¯(ξ)  ≥V0−κg(V0)U0∫0+∞e−κηdη  =V0−g(V0)U0  =[J−g(V0)]U0  ≥0.

The proof is completed.

**Lemma 6**

For *α* > 0 sufficiently small and *σ* > 0 sufficiently large, the function $\underline{U}(\xi )$ satisfies

(27) $$\begin{array}{c} {c\underline{U}}^{\prime }\leq -g\left(\bar{V}\left(\xi \right)\right)\underline{U}\left(\xi \right) \end{array}$$

for any *ξ* ≠ 1/*α*ln⁡(*U* ^0^/*σ*).

**Proof**

Let *σ* > 0 be sufficiently large to ensure 1/*α*ln⁡(*U* ^0^/*σ*) < ln⁡*V* ^0^/*λ*_1_. When *ξ* > 1/*α*ln⁡(*U* ^0^/*σ*), then $\underline{U}(\xi )=0$ and the lemma is obviously true. Now, suppose *ξ* < 1/*α*ln⁡(*U* ^0^/*σ*). Then, $\underline{U}(\xi )=U^{0}-\sigma e^{\alpha \xi }$ and

(28) −cU_′−g(V¯(ξ))U_(ξ) =cσαeαξ−[g′(0)V¯(ξ)+g′′(θV¯(ξ))2(V¯(ξ))2]  ×(U0−σeαξ) =cσαeαξ−g′(0)U0eλ1ξ+g′(0)V¯(ξ)σeαξ  −g′′(θV¯(ξ))2(V¯(ξ))2(U0−σeαξ) ≥eαξ[cσα−g′(0)U0e(λ1−α)(1/α)ln⁡(U0/σ)] =eαξ[cσα−g′(0)U0e(λ1−α)(U0σ)1/α],

where 0 < *θ* < 1. Let *σ* = 1/*α*. Since

(29) $$\begin{array}{c} \underset{\alpha \to 0^{+}}{\lim}{\left(\frac{U^{0}}{\sigma }\right)}^{1/\alpha }=\underset{\alpha \to 0^{+}}{\lim}{\left(U^{0}\alpha \right)}^{1/\alpha }=0, \end{array}$$

we can find *α* > 0 sufficiently small and *σ* > 0 sufficiently large such that

(30) $$\begin{array}{c} -{c\underline{U}}^{\prime }-g\left(\bar{V}\left(\xi \right)\right)\underline{U}\left(\xi \right)\geq 0. \end{array}$$

Thus, the proof is completed.

**Lemma 7**

Let *ε* = min⁡{*α*, *λ*_1_, *λ*_2_ − *λ*_1_}/2. Then, for *M* > 0 large enough, the function $\underline{V}(\xi )$ satisfies

(31) $$\begin{array}{c} {c\underline{V}}^{\prime }\leq {\underline{V}}^{\prime \prime }+\kappa \overset{+\infty }{\underset{0}{\int }}e^{-\kappa \eta }g\left(\underline{V}\left(\xi -c\eta \right)\right)\underline{U}\left(\xi -c\eta \right)d\eta -\underline{V}\left(\xi \right) \end{array}$$

for any *ξ* ≠ 1/*ε*ln⁡(1/*M*).

**Proof**

It is clear that $\underline{U}(\xi )=0$ if and only if *ξ* = 1/*α*ln⁡(*U* ^0^/*σ*), that $\underline{V}(\xi )=0$ if and only if *ξ* = 1/*ε*ln⁡(1/*M*), and that 1/*ε*ln⁡(1/*M*) < 1/*α*ln⁡(*U* ^0^/*σ*) if and only if *M* > (*σ*/*U* ^0^)^(*ε*/*α*)^. Let *M* > (*σ*/*U* ^0^)^(*ε*/*α*)^. When *ξ* > 1/*ε*ln⁡(1/*M*), then *e* ^*λ*_1_*ξ*^(1 − *Me* ^*εξ*^) < 0, $\underline{V}(\xi )=0$, and Lemma 7 holds.

In this paragraph, assume *ξ* < 1/*ε*ln⁡(1/*M*). Then, *ξ* < 1/*α*ln⁡(*U* ^0^/*σ*), $\underline{U}(\xi )=U^{0}-\sigma e^{\alpha \xi }>0$, and $\underline{V}(\xi )=e^{\lambda_{1}\xi }(1-Me^{\varepsilon \xi })>0$. To prove this lemma, it is enough to show

(32) 0≤e−λ1ξ[V_′′−cV_′+κ∫0+∞e−κηg(V_(ξ−cη))×U_(ξ−cη)dη−V_(ξ)]=λ12−M(λ1+ε)2eεξ−cλ1+cM(λ1+ε)eεξ−1 +Meεξ+e−λ1ξκ∫0+∞e−κη×[g′(0)V_(ξ−cη)+g′′(θV_(ξ−cη))2V_2(ξ−cη)]×U_(ξ−cη)dη=λ12−cλ1+κg′(0)U0∫0+∞e−(κ+cλ1)ηdη−1 +M[−(λ1+ε)2+c(λ1+ε)−κg′(0)U0∫0+∞e−(κ+c(λ1+ε))ηdη+1]eεξ −κg′(0)σeαξ∫0+∞e−(κ+cλ1+cα)ηdη +Mκg′(0)σe(α+ε)ξ∫0+∞e−(κ+cλ1+cε+cα)ηdη +κ2eλ1ξ∫0+∞e−(κ+2cλ1)ηg′′(θV_(ξ−cη))×(1−Meεξe−εcη)2U_(ξ−cη)dη=M−cH(λ1+ε)κ+cλ1+cεeεξ−κg′(0)σκ+cλ1+cαeαξ +Mκg′(0)σκ+cλ1+cε+cαe(α+ε)ξ +κ2eλ1ξ∫0+∞e−(κ+2cλ1)ηg′′(θV_(ξ−cη))×(1−Meεξe−εcη)2U_(ξ−cη)dη,

where 0 < *θ* < 1. Since

(33) 0≥κ2eλ1ξ∫0+∞e−(κ+2cλ1)ηg′′(θV_(ξ−cη))×(1−Meεξe−εcη)2U_(ξ−cη)dη≥−κ2eλ1ξ∫0+∞e−(κ+2cλ1)ηM¯0U0dη=−κM¯0U02(κ+2cλ1)eλ1ξ,

we only need to show

(34) M−cH(λ1+ε)κ+cλ1≥κg′(0)σκ+cλ1+cαe(α−ε)ξ +κM¯0U02(κ+2cλ1)e(λ1−ε)ξ.

Since *ξ* < 1/*α*ln⁡(*U* ^0^/*σ*), then

(35) $$\begin{array}{c} e^{(\alpha -\varepsilon )\xi }<{\left(U^{0}\sigma^{-1}\right)}^{(\alpha -\varepsilon )/\alpha },\\ e^{(\lambda_{1}-\varepsilon )\xi }<{\left(U^{0}\sigma^{-1}\right)}^{(\lambda_{1}-\varepsilon )/\alpha }. \end{array}$$

Since *H*(*λ*_1_ + *ε*) < 0, inequality (34) satisfies if

(36) M>−[κg′(0)σκ+cλ1+cα(U0σ−1)(α−ε)/α    +κM¯0U02(κ+2cλ1)(U0σ−1)(λ1−ε)/α]  ×κ+cλ1cH(λ1+ε).

The proof is completed.

To apply Schauder's fixed point theorem, we will introduce a topology in *C*(*R*, *R* ^2^). Let *μ* be a positive constant which will be specified in the following. For Φ(*ξ*) = (*ϕ*_1_(*ξ*), *ϕ*_2_(*ξ*)), define

(37) $$\begin{array}{c} {\left|\Phi \left(\cdot \right)\right|}_{\mu }=\max\left\{\underset{\xi \in R}{\sup}\left|\phi_{1}\left(\xi \right)\right|e^{-\mu |\xi |},\underset{\xi \in R}{\sup}\left|\phi_{2}\left(\xi \right)\right|e^{-\mu |\xi |}\right\},\\ B_{\mu }\left(R,R^{2}\right)=\left\{\Phi \left(\cdot \right)\in C\left(R,R^{2}\right):{\left|\Phi \left(\cdot \right)\right|}_{\mu }<+\infty \right\}. \end{array}$$

We will find traveling wave solutions in the following profile set:

(38) Γ={(U(·),V(·))∈C(R,R2):U_(ξ)≤U(ξ)≤U0,   V_(ξ)≤V(ξ)≤V¯(ξ)  for  any  ξ∈R}.

Obviously, Γ is closed and convex in *C*(*R*, *R* ^2^). Firstly, we change system (18) into the following form:

(39) $$\begin{array}{c} cU^{\prime }+\beta_{1}U\left(\xi \right)=H_{1}\left(U,V\right)\left(\xi \right),\\ -V^{\prime \prime }+{cV}^{\prime }+V\left(\xi \right)=H_{2}\left(U,V\right)\left(\xi \right), \end{array}$$

where *β*_1_ > lim⁡_*V*→+*∞*_ *g*(*V*),

(40) $$\begin{array}{c} H_{1}\left(U,V\right)\left(\xi \right)=\left[\beta_{1}-g\left(V\left(\xi \right)\right)\right]U\left(\xi \right),\\ H_{2}\left(U,V\right)\left(\xi \right)=\kappa \overset{+\infty }{\underset{0}{\int }}e^{-\kappa \eta }g\left(V\left(\xi -c\eta \right)\right)U\left(\xi -c\eta \right)d\eta . \end{array}$$

Suppose Λ_1_ < 0 < Λ_2_ are the two roots of equation Λ^2^ − *c*Λ − 1 = 0. Furthermore, define *F* = (*F*_1_, *F*_2_) : Γ → *C*(*R*, *R* ^2^) by

(41) F1(U(·),V(·))(ξ)=1c∫−∞ξe−(β1/c)(ξ−t)H1(U,V)(t)dt,F2(U(·),V(·))(ξ)=1Λ2−Λ1 ×[∫−∞ξeΛ1(ξ−t)H2(U,V)(t)dt   +∫ξ+∞eΛ2(ξ−t)H2(U,V)(t)dt].

In the remainder of this paper, it is always assumed that *μ* < min⁡{*β*_1_/*c*, *κ*/*c*, −Λ_1_, Λ_2_}.

**Lemma 8**

Consider *F*(Γ) ⊂ Γ.

**Proof**

Let (*U*(·), *V*(·)) ∈ Γ; that is $\underline{U}(\xi )\leq U(\xi )\leq U^{0}$, $\underline{V}(\xi )\leq V(\xi )\leq \bar{V}(\xi )$. Then, we need to prove that

(42) $$\begin{array}{c} \underline{U}\left(\xi \right)\leq F_{1}\left(U\left(\cdot \right),V\left(\cdot \right)\right)\left(\xi \right)\leq U^{0},\\ \underline{V}\left(\xi \right)\leq F_{2}\left(U\left(\cdot \right),V\left(\cdot \right)\right)\left(\xi \right)\leq \bar{V}\left(\xi \right). \end{array}$$

First of all, we have

(43) F1(U(·),V(·))(ξ)=1c∫−∞ξe−(β1/c)(ξ−t)H1(U,V)(t)dt,≤1c∫−∞ξe−(β1/c)(ξ−t)β1U0dt=U0.

From Lemma 6 and system (39), we get

(44) cU_′+β1U_(ξ)≤H1(U_,V¯)(ξ)=[β1−g(V¯(ξ))]U_(ξ)≤[β1−g(V(ξ))]U(ξ)=H1(U,V)(ξ),

where the second inequality is due to *β*_1_ > lim⁡_*V*→+*∞*_ *g*(*V*). Then,

(45) F1(U(·),V(·))(ξ)=1c∫−∞ξe−(β1/c)(ξ−t)H1(U,V)(t)dt,≥1c∫−∞ξe−(β1/c)(ξ−t)[cU_′(t)+β1U_(t)]dt=U_(ξ).

Therefore, we have proved $\underline{U}(\xi )\leq F_{1}(U(\cdot ),V(\cdot ))(\xi )\leq U^{0}$.

Now, we study *F*_2_(*U*(·), *V*(·))(*ξ*). If *ξ* ≥ *ξ*_0_≜1/*ε*ln⁡(1/*M*), then $\underline{V}(\xi )=0$, which implies that $F_{2}(U(\cdot ),V(\cdot ))(\xi )\geq \underline{V}(\xi )$ since $U(\xi )\geq \underline{U}(\xi )\geq 0$, $V(\xi )\geq \underline{V}(\xi )\geq 0$. Assume *ξ* < *ξ*_0_. From Lemma 7 and system (39), it is clear that

(46) −V_′′+cV_′+V_(ξ)≤H2(U_,V_)(ξ)=κ∫0+∞e−κηg(V_(ξ−cη))U_(ξ−cη)dη≤κ∫0+∞e−κηg(V(ξ−cη))U(ξ−cη)dη=H2(U,V)(ξ),

which implies

(47) F2(U(·),V(·))(ξ) =1Λ2−Λ1[∫−∞ξeΛ1(ξ−t)H2(U,V)(t)dt+∫ξ+∞eΛ2(ξ−t)H2(U,V)(t)dt] ≥1Λ2−Λ1∫−∞ξeΛ1(ξ−t)[−V_′′(t)+cV_′(t)+V_(t)]dt  +1Λ2−Λ1∫ξξ0eΛ2(ξ−t)[−V_′′(t)+cV_′(t)+V_(t)]dt  +1Λ2−Λ1∫ξ0+∞eΛ2(ξ−t)[−V_′′(t)+cV_′(t)+V_(t)]dt =V_(ξ)+1Λ2−Λ1eΛ2(ξ−ξ0)[V_′(ξ0+0)−V_′(ξ0−0)] ≥V_(ξ),

where the final inequality is due to ${\underline{V}}^{\prime }(\xi_{0}+0)=0$ and ${\underline{V}}^{\prime }(\xi_{0}-0)<0$. In conclusion, $F_{2}(U(\cdot ),V(\cdot ))(\xi )\geq \underline{V}(\xi )$ for any *ξ* ∈ *R*.

Similarly, we can show $F_{2}(U(\cdot ),V(\cdot ))(\xi )\leq \bar{V}(\xi )$ and the proof is completed.

**Lemma 9**

Map *F* = (*F*_1_, *F*_2_) : Γ → *C*(*R*, *R* ^2^) is continuous with respect to the norm |·|_*μ*_ in *B*_*μ*_(*R*, *R* ^2^).

**Proof**

For Φ_1_(·) = (*U*_1_(·), *V*_1_(·)), Φ_2_(·) = (*U*_2_(·), *V*_2_(·)) ∈ Γ, we have

(48) |H1(U1,V1)(ξ)−H1(U2,V2)(ξ)|e−μ|ξ|≤β1|U1(ξ)−U2(ξ)|e−μ|ξ| +|g(V1(ξ))U1(ξ)−g(V2(ξ))U2(ξ)|e−μ|ξ|≤β1|U1(·)−U2(·)|μ +|g(V1(ξ))[U1(ξ)−U2(ξ)]+U2(ξ)[g(V1(ξ))−g(V2(ξ))]|e−μ|ξ|≤β1|U1(·)−U2(·)|μ+J|U1(ξ)−U2(ξ)|e−μ|ξ| +U0g′(V∗)|V1(ξ)−V2(ξ)|e−μ|ξ|≤(β1+J)|U1(·)−U2(·)|μ+U0g′(0)|V1(·)−V2(·)|μ≤M1|Φ1(·)−Φ2(·)|μ,

where *M*_1_ = *β*_1_ + *J* + *U* ^0^ *g*′(0), and V* is between *V*_1_(*ξ*) and *V*_2_(*ξ*). Therefore, we have

(49) $$\begin{array}{c} {\left|H_{1}\left(\Phi_{1}\left(\cdot \right)\right)-H_{1}\left(\Phi_{2}\left(\cdot \right)\right)\right|}_{\mu }\leq M_{1}{\left|\Phi_{1}\left(\cdot \right)-\Phi_{2}\left(\cdot \right)\right|}_{\mu }. \end{array}$$

Then,

(50) |F1(U1(·),V1(·))(ξ)−F1(U2(·),V2(·))(ξ)|e−μ|ξ| ≤1ce−μ|ξ|∫−∞ξe−(β1/c)(ξ−t)×|H1(U1,V1)(t)−H1(U2,V2)(t)|dt =1ce−μ|ξ|−(β1/c)ξ∫−∞ξe(β1/c)t+μ|t|×|H1(U1,V1)(t)−H1(U2,V2)(t)|e−μ|t|dt ≤M1|Φ1(·)−Φ2(·)|μ1ce−μ|ξ|−(β1/c)ξ∫−∞ξe(β1/c)t+μ|t|dt.

Thus, when *ξ* ≤ 0, we get

(51) |F1(U1(·),V1(·))(ξ)−F1(U2(·),V2(·))(ξ)|e−μ|ξ| ≤M1|Φ1(ξ)−Φ2(ξ)|μ1ce(μ−β1/c)ξ∫−∞ξe(β1/c−μ)tdt =M1β1−cμ|Φ1(·)−Φ2(·)|μ.

When *ξ* > 0, it follows that

(52) |F1(U1(·),V1(·))(ξ)−F1(U2(·),V2(·))(ξ)|e−μ|ξ| ≤M1|Φ1(·)−Φ2(·)|μ1ce−μ|ξ|−(β1/c)ξ∫−∞ξe(β1/c)t+μ|t|dt =M1|Φ1(·)−Φ2(·)|μ1ce−μξ−(β1/c)ξ  ×[∫−∞0e(β1/c)t−μtdt+∫0ξe(β1/c)t+μtdt] =M1|Φ1(·)−Φ2(·)|μ1ce−μξ−(β1/c)ξ  ×[2c2μβ12−c2μ2+cβ1+cμe(β1/c+μ)ξ] ≤M1(2cμβ12−c2μ2+1β1+cμ)|Φ1(·)−Φ2(·)|μ.

Consequently, we have proved that

(53) |F1(U1(·),V1(·))(ξ)−F1(U2(·),V2(·))(ξ)|e−μ|ξ|  ≤M2|Φ1(·)−Φ2(·)|μ

for any *ξ* ∈ *R*, where

(54) $$\begin{array}{c} M_{2}=\max\left\{\frac{M_{1}}{\beta_{1}-c\mu },M_{1}\left(\frac{2c\mu }{\beta_{1}^{2}-c^{2}\mu^{2}}+\frac{1}{\beta_{1}+c\mu }\right)\right\}. \end{array}$$

That is,

(55) $$\begin{array}{c} {\left|F_{1}\left(\Phi_{1}\left(\cdot \right)\right)-F_{1}\left(\Phi_{2}\left(\cdot \right)\right)\right|}_{\mu }\leq M_{2}{\left|\Phi_{1}(\cdot )-\Phi_{2}(\cdot )\right|}_{\mu }. \end{array}$$

In conclusion, *F*_1_ : Γ → *C*(*R*, *R*) is continuous with respect to the norm |·|_*μ*_ in *B*_*μ*_(*R*, *R*).

In addition, consider *F*_2_(*U*(*ξ*), *V*(*ξ*)). Firstly, we have

(56) |H2(U1,V1)(ξ)−H2(U2,V2)(ξ)|e−μ|ξ| ≤κe−μ|ξ|∫0+∞e−κη|g(V1(ξ−cη))U1(ξ−cη)−g(V2(ξ−cη))U2(ξ−cη)|dη ≤κe−μ|ξ|∫0+∞e−κη[J|U1(ξ−cη)−U2(ξ−cη)|+U0g′(0)×|V1(ξ−cη)−V2(ξ−cη)|]dη ≤[J|U1(·)−U2(·)|μ+U0g′(0)|V1(ξ)−V2(ξ)|μ]  ×κe−μ|ξ|∫0+∞e−κη+μ|ξ−cη|dη ≤|Φ1(·)−Φ2(·)|μ  ×[(J+U0g′(0))κ∫0+∞e−μ|ξ|−κη+μ|ξ−cη|dη] ≤|Φ1(·)−Φ2(·)|μ  ×[(J+U0g′(0))κ∫0+∞e−(κ−cμ)ηdη] =M3|Φ1(·)−Φ2(·)|μ,

where *M*_3_ = (*J* + *U* ^0^ *g*′(0))*κ*/(*κ* − *cμ*). Therefore,

(57) |F2(U1(·),V1(·))(ξ)−F2(U2(·),V2(·))(ξ)|e−μ|ξ|≤e−μ|ξ|Λ2−Λ1 ×[∫−∞ξeΛ1(ξ−t)|H2(U1,V1)(t)−H2(U2,V2)(t)|dt+∫ξ+∞eΛ2(ξ−t)|H2(U1,V1)(t)−H2(U2,V2)(t)|dt]≤M3e−μ|ξ|Λ2−Λ1 ×[∫−∞ξeΛ1(ξ−t)+μ|t|dt+∫ξ+∞eΛ2(ξ−t)+μ|t|dt] ×|Φ1(·)−Φ2(·)|μ.

If *ξ* < 0, it holds that

(58) |F2(U1(·),V1(·))(ξ)−F2(U2(·),V2(·))(ξ)|e−μ|ξ|≤M3eμξΛ2−Λ1 ×[eΛ1ξ∫−∞ξe−(Λ1+μ)tdt+eΛ2ξ∫ξ0e−(Λ2+μ)tdt+eΛ2ξ∫0+∞e(μ−Λ2)tdt]|Φ1(·)−Φ2(·)|μ=M3Λ2−Λ1 ×[1−Λ1−μ+1−e(Λ2+μ)ξΛ2+μ+e(Λ2+μ)ξΛ2−μ] ×|Φ1(·)−Φ2(·)|μ≤M3Λ2−Λ1 ×(1−Λ1−μ+1Λ2+μ+1Λ2−μ)|Φ1(·)−Φ2(·)|μ.

If *ξ* ≥ 0, we have

(59) |F2(U1(·),V1(·))(ξ)−F2(U2(·),V2(·))(ξ)|e−μ|ξ|≤M3e−μξΛ2−Λ1 ×[eΛ1ξ∫−∞0e−(Λ1+μ)tdt+eΛ1ξ∫0ξe(μ−Λ1)tdt+eΛ2ξ∫ξ+∞e(μ−Λ2)tdt]|Φ1(·)−Φ2(·)|μ=M3Λ2−Λ1[e(Λ1−μ)ξ−Λ1−μ+1−e(Λ1−μ)ξμ−Λ1+1Λ2−μ] ×|Φ1(·)−Φ2(·)|μ≤M3Λ2−Λ1(1−Λ1−μ+1μ−Λ1+1Λ2−μ) ×|Φ1(·)−Φ2(·)|μ.

Consequently, we conclude that

(60) $$\begin{array}{c} {\left|F_{2}\left(\Phi_{1}\left(\cdot \right)\right)-F_{2}\left(\Phi_{2}\left(\cdot \right)\right)\right|}_{\mu }\leq M_{4}{\left|\Phi_{1}\left(\cdot \right)-\Phi_{2}\left(\cdot \right)\right|}_{\mu }, \end{array}$$

where

(61) M4=M3Λ2−Λ1 ×max⁡{1−Λ1−μ+1Λ2+μ+1Λ2−μ,1−Λ1−μ+1μ−Λ1+1Λ2−μ}.

Thus, *F*_2_ : Γ → *C*(*R*, *R*) is continuous with respect to the norm |·|_*μ*_ in *B*_*μ*_(*R*, *R*). The proof is completed.

**Lemma 10**

Map *F* = (*F*_1_, *F*_2_) : Γ → Γ is compact with respect to the norm |·|_*μ*_ in *B*_*μ*_(*R*, *R* ^2^).

**Proof**

For any Φ(*ξ*) = (*U*(*ξ*), *V*(*ξ*)) ∈ Γ, it is clear that

(62) ddξF1(Φ(·))(ξ)=1cH1(Φ(·))(ξ) −β1c2∫−∞ξe−(β1/c)(ξ−t)H1(Φ(·))(t)dt.

Since |*H*_1_(*U*, *V*)(*ξ*)| = |[*β*_1_ − *g*(*V*(*ξ*))]*U*(*ξ*)| ≤ *β*_1_ *U* ^0^, we have

(63) |ddξF1(Φ(·))(ξ)|e−μ|ξ|  ≤|ddξF1(Φ(·))(ξ)|  ≤β1U0c+β1c2e−(β1/c)ξ∫−∞ξe(β1/c)tβ1U0dt  =2β1U0c,

which implies that  |(*d*/*dξ*)*F*_1_(Φ(·))(·)|_*μ*_ < 2*β*_1_ *U* ^0^/*c*. Since Φ(·) = (*U*(·), *V*(·)) ∈ Γ, we get

(64) |H2(U,V)(ξ)|=κ∫0+∞e−κηg(V(ξ−cη))U(ξ−cη)dη≤κ∫0+∞e−κηβ1U0dη+V0=β1U0+V0.

Then,

(65) |ddξF2(Φ(·))(ξ)|  =1Λ2−Λ1|Λ1∫−∞ξeΛ1(ξ−t)H2(U,V)(t)dt+Λ2∫ξ+∞eΛ2(ξ−t)H2(U,V)(t)dt|  ≤β1U0+V0Λ2−Λ1   ×[|Λ1|∫−∞ξeΛ1(ξ−t)dt+Λ2∫ξ+∞eΛ2(ξ−t)dt]  =2(β1U0+V0)Λ2−Λ1,

which implies that|(*d*/*dξ*)*F*_2_(Φ(·))(·)|_*μ*_ < 2(*β*_1_ *U* ^0^ + *V* ^0^)/(Λ_2_ − Λ_1_). Consequently, |(*d*/*dξ*)*F*_1_(Φ(·))(·)|_*μ*_ and |(*d*/*dξ*)*F*_2_(Φ(·))(·)|_*μ*_ are bounded, which shows that *F*(Γ) is uniformly bounded and equicontinuous with respect to the norm |·|_*μ*_ in *B*_*μ*_(*R*, *R* ^2^).

Furthermore, for any positive integer *n*, define

(66) Fn(U(·),V(·))(ξ)  ={F(U(·),V(·))(ξ),ξ∈[−n,n],F(U(·),V(·))(−n),ξ∈(−∞,−n],F(U(·),V(·))(n),ξ∈[n,+∞].

Obviously, for fixed *n*, *F* ^*n*^(Γ) is uniformly bounded and equicontinuous with respect to the norm |·|_*μ*_ in *B*_*μ*_(*R*, *R* ^2^), which implies that *F* ^*n*^ : Γ → Γ is a compact operator. Since

(67) |F1(U(·),V(·))(ξ)|≤1c∫−∞ξe−(β1/c)(ξ−t)β1U0dt=U0,|F2(U(·),V(·))(ξ)|≤β1U0+V0Λ2−Λ1 ×{∫−∞ξeΛ1(ξ−t)dt+∫ξ+∞eΛ2(ξ−t)dt}=β1U0+V0|Λ1|Λ2,

we have that

(68) |F1n(U(·),V(·))(·)−F1(U(·),V(·))(·)|μ =sup⁡ξ∈R|F1n(U(·),V(·))(ξ)−F1(U(·),V(·))(ξ)|e−μ|ξ| =sup⁡ξ∈(−∞,−n]∪[n,+∞)|F1n(U(·),V(·))(ξ)−F1(U(·),V(·))(ξ)|e−μ|ξ| ≤2U0e−μn⟶0, as  n⟶+∞.

Similarly, we can prove that |*F*_2_^*n*^(*U*(·),*V*(·))(·)−*F*_2_(*U*(·),*V*(·))(·)|_*μ*_ → 0 when *n* → +*∞*. Thus, |*F* ^*n*^(*U*(·),*V*(·))(·)−*F*(*U*(·),*V*(·))(·)|_*μ*_ → 0 when *n* → +*∞*. By Proposition 2.1 in Zeilder [40], we have that *F* ^*n*^ converges to *F* in Γ with respect to the norm |·|_*μ*_. Consequently, *F* : Γ → Γ is compact with respect to the norm |·|_*μ*_. The proof is completed.

**Proof of Theorem 1**

Combination of Schauder's fixed point theorem and Lemmas 8, 9, and 10 shows that there exists a nonnegative traveling wave solution (*U*_*c*_(·), *V*_*c*_(·)) ∈ Γ such that (*U*_*c*_(*ξ*), *V*_*c*_(*ξ*)) → (*U* ^0^, 0) when *ξ* → −*∞*. By the definition of $\underline{U}(\xi )$ and $\underline{V}(\xi )$, there is a *ξ*_0_ < 0 such that $\underline{U}(\xi )>0$ and $\underline{V}\left(\xi \right)>0$ when *ξ* < *ξ*_0_. Therefore, if *ξ* < *ξ*_0_, we have that *cU*_*c*_′ = −*g*(*V*_*c*_(*ξ*))*U*(*ξ*) < 0 which implies that *U* ^0^ > *U* ^1^ ≥ 0. It is clear that *U*_*c*_(*ξ*) is nonincreasing in *R*.

From Lemma 2 and system (18), it follows that

(69) cUc′=−g(Vc(ξ))Uc(ξ),Wc(ξ)=κ∫0+∞e−κηg(Vc(ξ−cη))Uc(ξ−cη)dη,cVc′=Vc′′+Wc(ξ)−Vc(ξ).

By the first and second equation of (69), we have

(70) ∫−∞+∞Wc(ξ)dξ  =−κc∫−∞+∞∫0+∞e−κηUc′(ξ−cη)dη dξ  =−κc∫0+∞e−κη∫−∞+∞Uc′(ξ−cη)dξ dη  =(U0−U1)κc∫0+∞e−κηdη  =c(U0−U1).

Since the traveling wave solution (*U*_*c*_(·), *V*_*c*_(·)) ∈ Γ and is the fixed point of the operator *F*, thus (*U*_*c*_(*ξ*), *V*_*c*_(*ξ*)) = *F*(*U*_*c*_(·), *V*_*c*_(·))(*ξ*). The proof of Lemma 10 shows that *V*_*c*_′(*ξ*) = *F*_2_′(*U*_*c*_(·), *V*_*c*_(·))(*ξ*) is bounded. Using L'Hopital principal shows that

(71) $$\begin{array}{c} V_{c}^{\prime }\left(-\infty \right)=F_{2}^{\prime }\left(U_{c}\left(\cdot \right),V_{c}\left(\cdot \right)\right)\left(-\infty \right)=0. \end{array}$$

Integrating the third equation of (69) from −*∞* to *ξ* yields

(72) $$\begin{array}{c} cV_{c}\left(\xi \right)=V_{c}^{\prime }\left(\xi \right)+\overset{\xi }{\underset{-\infty }{\int }}W_{c}\left(\eta \right)d\eta -\overset{\xi }{\underset{-\infty }{\int }}V_{c}\left(\eta \right)d\eta . \end{array}$$

Then, the boundedness of *V*_*c*_(*ξ*) and *V*_*c*_′(*ξ*) implies ∫_−*∞*_^+*∞*^ *V*_*c*_(*η*)*dη* < +*∞*, which shows that lim⁡_*ξ*→+*∞*_ *V*_*c*_(*ξ*) = 0 and ∫_−*∞*_^+*∞*^ *W*_*c*_(*η*)*dη* = ∫_−*∞*_^+*∞*^ *V*_*c*_(*η*)*dη* = *c*(*U* ^0^ − *U* ^1^).

Next, we prove 0 ≤ *V*_*c*_(*ξ*) ≤ *U* ^0^ − *U* ^1^, which is motivated by Wang and Wu [34]. Define

(73) $$\begin{array}{c} R\left(\xi \right)=\frac{1}{c}\overset{\xi }{\underset{-\infty }{\int }}V_{c}\left(\eta \right)d\eta +\frac{1}{c}\overset{+\infty }{\underset{\xi }{\int }}e^{c(\xi -\eta )}V_{c}\left(\eta \right)d\eta . \end{array}$$

It is clear that *R*(*ξ*) satisfies

(74) $$\begin{array}{c} cR^{\prime }=R^{\prime \prime }+R\left(\xi \right), \end{array}$$

*R*(−*∞*) = 0, and *R*(+*∞*) = *U* ^0^ − *U* ^1^. Denote *N*(*ξ*) = *V*_*c*_(*ξ*) + *R*(*ξ*), which satisfies

(75) $$\begin{array}{c} cN^{\prime }=N^{\prime \prime }+W_{c}\left(\xi \right). \end{array}$$

Due to *N*′(+*∞*) = 0, it follows that

(76) $$\begin{array}{c} N^{\prime }\left(\xi \right)=\overset{+\infty }{\underset{\xi }{\int }}e^{c(\xi -\eta )}W_{c}\left(\eta \right)d\eta \geq 0, \end{array}$$

which implies that *N*(*ξ*) is nondecreasing in *R*. Since *N*(−*∞*) = 0 and *N*(+*∞*) = *U* ^0^ − *U* ^1^, we have 0 ≤ *V*_*c*_(*ξ*) ≤ *U* ^0^ − *U* ^1^ for all *ξ* ∈ *R*. The proof is completed.

## 3. Nonexistence of Traveling Wave Solutions

In this section, we give the conditions on which system (5) has no traveling wave solutions.

**Theorem 11**

(I) Assume *U* ^0^ > 1/*g*′(0). Then, for any 0 < *c* < c*, system (5) has no nonnegative nontrivial traveling wave solutions (*U*(*y* + *cτ*), *W*(*y* + *cτ*), *V*(*y* + *cτ*)) satisfying boundary condition (8).

(II) Suppose 0 < *U* ^0^ ≤ 1/*g*′(0). Then, for any *c* > 0, system (5) has no traveling wave solutions (*U*(*y* + *cτ*), *W*(*y* + *cτ*), *V*(*y* + *cτ*)) satisfying boundary condition (8).

To prove (I) by two-sided Laplace transform, the following lemma about the exponential decrease of traveling wave solutions is needed.

**Lemma 12**

Assume *U* ^0^ > 1/*g*′(0). If (*U*(*y* + *cτ*), *W*(*y* + *cτ*), *V*(*y* + *cτ*)) is a nonnegative nontrivial traveling wave solution of (5) satisfying boundary condition (8), there exists a positive constant *α* such that

(77) $$\begin{array}{c} \underset{\xi \in R}{\sup}\left\{W\left(\xi \right)e^{-\alpha \xi }\right\}<+\infty ,\;\;\underset{\xi \in R}{\sup}\left\{V\left(\xi \right)e^{-\alpha \xi }\right\}<+\infty . \end{array}$$

**Proof**

Substituting traveling wave profile

(78) $$\begin{array}{c} u_{2}\left(y,\tau \right)=W\left(\xi \right),\;\;u_{3}\left(y,\tau \right)=V\left(\xi \right),\;\;\xi =y+c\tau \end{array}$$

into the second and third equations of (5), it follows that

(79) $$\begin{array}{c} cW^{\prime }=\kappa \left[g\left(V\right)U-W\right],\\ cV^{\prime }=V^{\prime \prime }+W-V. \end{array}$$

Setting *V*′ = *Z*, we have

(80) $$\begin{array}{c} cW^{\prime }=\kappa \left[g\left(V\right)U-W\right],\\ V^{\prime }=Z,\\ Z^{\prime }=cZ+V-W. \end{array}$$

Furthermore, system (80) can be written as

(81) $$\begin{array}{c} \psi^{\prime }=A\psi +f\left(\xi ,\psi \right), \end{array}$$

where

(82) $$\begin{array}{c} A=\left[\begin{array}{ccc} -\frac{\kappa }{c} & \frac{\kappa g^{\prime }\left(0\right)U^{0}}{c} & 0\\ 0 & 0 & 1\\ -1 & 1 & c \end{array}\right],\\ \psi =\left[\begin{array}{c} W\\ V\\ Z \end{array}\right],\\ f\left(\xi ,\psi \right)=\left[\begin{array}{c} \frac{\kappa U^{0}\bar{G}\left(V\right)}{c}+\frac{\kappa \left(U-U^{0}\right)g\left(V\right)}{c}\\ 0\\ 0 \end{array}\right], \end{array}$$

and $\bar{G}(V)=g(V)-g^{\prime }(0)V$. Obviously, ${\bar{G}}^{\prime }(0)=0$. Since

(83) $$\begin{array}{c} \underset{\xi \to -\infty }{\lim}U\left(\xi \right)=U^{0},\;\;\underset{\xi \to -\infty }{\lim}\psi \left(\xi \right)=0, \end{array}$$

and *g*′(*V*) is bounded in [0, +*∞*), for any small constant *ε* > 0, there exists *ξ*_0_ ∈ *R* such that

(84) $$\begin{array}{c} ||f\left(\xi ,\psi_{1}\right)-f\left(\xi ,\psi_{2}\right)||\leq \varepsilon ||\psi_{1}-\psi_{2}|| \end{array}$$

for all *ξ* ≤ *ξ*_0_.

Since (*U*(*ξ*), *W*(*ξ*), *V*(*ξ*)) is the solution of (79) if and only if (*U*(*ξ* − *η*), *W*(*ξ* − *η*), *V*(*ξ* − *η*)) is the solution of (79) for any *η* ∈ *R* and they satisfy the same boundary condition at ±*∞*, we can select large *η* such that (84) holds with *ξ*_0_ = 0 and (*U*(*ξ*), *W*(*ξ*), *V*(*ξ*)) being replaced by (*U*(*ξ* − *η*), *W*(*ξ* − *η*), *V*(*ξ* − *η*)). Therefore, we suppose *ξ*_0_ = 0.

Calculations show that the eigenfunction of *A* is *H*(*λ*) defined by (21). Then, by Lemma 4, there exists constant matrix *C* such that

(85) $$\begin{array}{c} C^{-1}AC=B=\left[\begin{array}{cc} \lambda_{0} & 0\\ 0 & Q \end{array}\right], \end{array}$$

where *λ*_0_ < 0 and the eigenvalues *λ*_1_, *λ*_2_ of 2 × 2 matrix *Q* have positive real parts. Setting *ψ* = *Cφ*, the system (81) then has the form

(86) $$\begin{array}{c} \frac{d\varphi }{d\xi }=B\varphi +G\left(\xi ,\varphi \right). \end{array}$$

Obviously, lim⁡_*ξ*→−*∞*_ *φ*(*ξ*) = 0 and *G*(*ξ*, *φ*) = *C* ^−1^ *f*(*ξ*, *Cψ*) satisfies (84). Denote

(87) $$\begin{array}{c} \Phi_{1}\left(\xi \right)=\left[\begin{array}{cc} e^{\lambda_{0}\xi } & 0\\ 0 & 0 \end{array}\right],\;\;\Phi_{2}\left(\xi \right)=\left[\begin{array}{cc} 0 & 0\\ 0 & e^{Q\xi } \end{array}\right]. \end{array}$$

Then, Φ_1_′ = *B*Φ_1_, Φ_2_′ = *B*Φ_2_, and

(88) $$\begin{array}{c} e^{B\xi }=\Phi_{1}\left(\xi \right)+\Phi_{2}\left(\xi \right). \end{array}$$

It is not difficult to see that we can choose positive constants *α* and *L* such that

(89) $$\begin{array}{c} ||\Phi_{1}\left(\xi \right)||\leq Le^{-2\alpha \xi }\;\text{for}\;\;\text{any}\;\;\xi \geq 0,\\ ||\Phi_{2}\left(\xi \right)||\leq Le^{2\alpha \xi }\;\text{for}\;\;\text{any}\;\;\xi \leq 0. \end{array}$$

It is clear that (86) is equivalent to

(90) $$\begin{array}{c} u_{1}\left(\xi \right)=e^{\lambda_{0}\xi }u_{1}\left(0\right)+\overset{\xi }{\underset{0}{\int }}e^{\lambda_{0}(\xi -\eta )}G_{1}\left(\eta ,u\left(\eta \right)\right)d\eta ,\\ u_{2}\left(\xi \right)=e^{Q\xi }u_{2}\left(0\right)+\overset{\xi }{\underset{0}{\int }}e^{Q(\xi -\eta )}G_{2}\left(\eta ,u\left(\eta \right)\right)d\eta , \end{array}$$

where

(91) $$\begin{array}{c} u={\left(u_{1},u_{2}^{T}\right)}^{T}=\varphi ,\;\;u_{1}=\varphi_{1},\;\;u_{2}={\left(\varphi_{2},\varphi_{3}\right)}^{T},\\ {\left(G_{1}\left(\eta ,u\right),G_{2}{\left(\eta ,u\right)}^{T}\right)}^{T}=G\left(\eta ,u\right). \end{array}$$

Since lim⁡_*ξ*→−*∞*_ *u*(*ξ*) = 0, then multiplying the first equality of (90) by *e* ^−*λ*_0_*ξ*^ and setting *ξ* → −*∞* yield

(92) $$\begin{array}{c} u_{1}\left(0\right)+\overset{-\infty }{\underset{0}{\int }}e^{-\lambda_{0}\eta }G_{1}\left(\eta ,u\left(\eta \right)\right)d\eta =0 \end{array}$$

or

(93) $$\begin{array}{c} u_{1}\left(0\right)=\overset{0}{\underset{-\infty }{\int }}e^{-\lambda_{0}\eta }G_{1}\left(\eta ,u\left(\eta \right)\right)d\eta . \end{array}$$

Thus, (90) has the form

(94) u(ξ)=∫−∞ξΦ1(ξ−η)G(η,u(η))dη+Φ2(ξ)u0−∫ξ0Φ2(ξ−η)G(η,u(η))dη.

where *u*_0_ = (0,*u*_2_(0)^*T*^)^*T*^.

To study the properties of *u*(*ξ*), we need to construct a functional sequence. Define

(95) u0(ξ)=0,uj+1(ξ)=∫−∞ξΦ1(ξ−η)G(η,uj(η))dη+Φ2(ξ)u0 −∫ξ0Φ2(ξ−η)G(η,uj(η))dη

for *ξ* ≤ 0. Obviously, we have

(96) $$\begin{array}{c} ||u^{1}\left(\xi \right)-u^{0}\left(\xi \right)||\leq ||\Phi_{2}\left(\xi \right)u_{0}||\leq L||u_{0}||e^{\alpha \xi } \end{array}$$

for any *ξ* ≤ 0. Assume that the induction hypothesis

(97) $$\begin{array}{c} ||u^{j}\left(\xi \right)-u^{j-1}\left(\xi \right)||\leq \frac{L||u_{0}||e^{\alpha \xi }}{2^{j-1}} \end{array}$$

holds for *j* = 1,2,…, *m* and *ξ* ≤ 0. Then, using the condition (84) satisfied by the function *G*, it follows from the induction hypothesis that for *ξ* ≤ 0 we have

(98) ||um+1(ξ)−um(ξ)|| ≤∫−∞ξ||Φ1(ξ−η)||ε||um(η)−um−1(η)||dη  +∫ξ0||Φ2(ξ−η)||ε||um(η)−um−1(η)||dη ≤ε∫−∞ξLe−2α(ξ−η)L||u0||eαη2m−1dη  +ε∫ξ0Le2α(ξ−η)L||u0||eαη2m−1dη ≤εL2||u0||eαξ3α2m−1+εL2||u0||eαξα2m−1 =8εL3αL||u0||eαξ2m.

Setting *ε* ≤ 3*α*/(8*L*) yields

(99) $$\begin{array}{c} ||u^{m+1}\left(\xi \right)-u^{m}\left(\xi \right)||\leq \frac{L||u_{0}||e^{\alpha \xi }}{2^{m}}. \end{array}$$

It then follows by induction that (97) holds for all *j* = 1,2, 3,… and *ξ* < 0. Thus, for any *n* > *m* > *N* and *ξ* < 0, we have

(100) ||un(ξ)−um(ξ)||≤∑j=N∞||uj+1(ξ)−uj(ξ)||≤L||u0||∑j=N∞12j=L||u0||2N−1,

which implies that {*u* ^*j*^(*ξ*)} is a Cauchy sequence of continuous functions. It follows that

(101) $$\begin{array}{c} \underset{j\to \infty }{\lim}u^{j}\left(\xi \right)=\bar{u}\left(\xi \right) \end{array}$$

uniformly for all *ξ* < 0. Taking the limit of both sides of (95), it follows from the uniform convergence that the continuous function $\bar{u}(\xi )$ satisfies the integral equation (94). Then, the uniqueness of solution of (94) implies $u(\xi )=\bar{u}(\xi )$. Consequently, (97) and

(102) $$\begin{array}{c} u^{m}\left(\xi \right)=\overset{m}{\underset{j=1}{\sum }}\left[u^{j}\left(\xi \right)-u^{j-1}\left(\xi \right)\right] \end{array}$$

show that

(103) $$\begin{array}{c} ||u\left(\xi \right)||\leq 2L||u_{0}||e^{\alpha \xi } \end{array}$$

for any *ξ* < 0. Since lim⁡_*ξ*→+*∞*_ *u*(*ξ*) = 0, it follows ||*u*(*ξ*)*e* ^−*αξ*^|| < +*∞*. Thus,

(104) $$\begin{array}{c} \underset{\xi \in R}{\sup}\left\{I_{u}\left(\xi \right)e^{-\alpha \xi }\right\}<+\infty ,\;\;\;\underset{\xi \in R}{\sup}\left\{I_{t}\left(\xi \right)e^{-\alpha \xi }\right\}<+\infty \end{array}$$

and the proof is completed.

**Proof of Theorem 11(I)**

Assume (5) has a traveling wave solution (*U*(*y* + *cτ*), *W*(*y* + *cτ*), *V*(*y* + *cτ*)) satisfying boundary condition (8). From the first equation of (18), it follows

(105) $$\begin{array}{c} U\left(\xi \right)=U^{0}e^{-(1/c)\int_{-\infty }^{\xi }g(V(\eta ))d\eta },\\ U^{0}-U\left(\xi \right)=U^{0}\left[1-e^{-(1/c)\int_{-\infty }^{\xi }g(V(\eta ))d\eta }\right]. \end{array}$$

By Lemma 12, there exists a positive constant *α*′ such that

(106) $$\begin{array}{c} \underset{\xi \in R}{\sup}\left\{\left[U^{0}-U\left(\xi \right)\right]e^{-\alpha^{\prime }\xi }\right\}<+\infty . \end{array}$$

For 0 < *λ* < *α*, define two-sided Laplace transform as follows:

(107) $$\begin{array}{c} L\left[V\left(\cdot \right)\right]\left(\lambda \right):=\overset{+\infty }{\underset{-\infty }{\int }}e^{-\lambda \xi }V\left(\xi \right)d\xi . \end{array}$$

Obviously, there exists λ* > 0 and *L*[*V*(·)](*λ*) is increasing in [0, λ*) such that λ* < +*∞* satisfying lim⁡_*λ*→*λ*^∗−^_ *L*[*V*(·)](*λ*) = +*∞* or λ* = +*∞*.

Furthermore, we have

(108) L[∫0+∞e−κηJ(V(·−cη))dη](λ)  =∫0+∞e−κη∫−∞+∞e−λξJ(V(·−cη))dξ dη  =∫0+∞e−κη∫−∞+∞e−λ(t+cη)J(V(t))dt dη  =∫0+∞e−(κ+cλ)η∫−∞+∞e−λtJ(V(t))dt dη  =1κ+cλL[J(V(·))](λ).

The second equality of (18) can be rewritten as

(109) $$\begin{array}{c} L\left[V\left(\cdot \right)\right]\left(\xi \right)=\overset{+\infty }{\underset{0}{\int }}\kappa e^{-\kappa \eta }Q\left(\xi -c\eta \right)d\eta , \end{array}$$

where

(110) L[V(·)](ξ)≔V′′(ξ)−cV′(ξ)+κg′(0)U0 ×∫0+∞e−κηV(ξ−cη)dη−V(ξ),Q(ξ):=[g′(0)V(ξ)U0−g(V(ξ))U(ξ)].

Denote *G*(*V*): = *g*′(0)*V* − *g*(*V*), from which it follows that *G*′(0) = 0. Since *g*(*V*) satisfies hypothesis (A1), we have *G*(*V*) ≥ 0 for any *V* ≥ 0.

Taking two-sided Laplace transform of (109), we have

(111) $$\begin{array}{c} \Theta \left(\lambda \right)L\left[V\left(\cdot \right)\right]\left(\lambda \right)=\frac{\kappa }{\kappa +c\lambda }L\left[Q\left(\cdot \right)\right]\left(\lambda \right). \end{array}$$

If λ* < +*∞*, it is clear that *L*[*V*(·)](λ*−) = +*∞*. Since

(112) $$\begin{array}{c} Q\left(\xi \right)=g^{\prime }\left(0\right)V\left(\xi \right)\left[U^{0}-U\left(\xi \right)\right]+G\left(V\left(\xi \right)\right)U\left(\xi \right), \end{array}$$

Lemma 12, inequality (106), and *G*′(0) = 0 imply *L*[*Q*(·)](λ*) < +*∞*. However, Lemma 4 shows that 0 < Θ(λ*)<+*∞*, which contradicts (111). Hence, we have λ* = +*∞*. Furthermore, (111) can be rewritten as

(113) $$\begin{array}{c} \overset{+\infty }{\underset{-\infty }{\int }}e^{-\lambda \xi }\left[\Theta \left(\lambda \right)V\left(\xi \right)-\overset{+\infty }{\underset{0}{\int }}\kappa e^{-\kappa \eta }Q\left(\xi -c\eta \right)d\eta \right]d\xi =0. \end{array}$$

Since lim⁡_*λ*→+*∞*_Θ(*λ*) = +*∞*, it is impossible that the above equality holds, again a contradiction.

**Proof of Theorem 11(II)**

Suppose (*U*(*y* + *cτ*), *W*(*y* + *cτ*), *V*(*y* + *cτ*)) is a traveling wave solution of system (5) satisfying boundary condition (8). Then, by the proof of Theorem 1, it is clear that ∫_−*∞*_^+*∞*^ *V*(*ξ*)*dξ* = ∫_−*∞*_^+*∞*^ *H*_2_(*U*, *V*)(*ξ*)*dξ*. Furthermore,

(114) ∫−∞+∞H2(U,V)(ξ)dξ  =∫−∞+∞∫0+∞κe−κηg(V(ξ−cη))U(ξ−cη)dη dξ  <∫−∞+∞∫0+∞κe−κηg′(0)V(ξ−cη)U0dη dξ  =g′(0)U0∫0+∞κe−κη∫−∞+∞V(ξ−cη)dξ dη  ≤∫0+∞κe−κη∫−∞+∞V(ξ)dξ dη  =∫−∞+∞V(ξ)dξ,

which is a contradiction. The proof is completed.
